# Topical application of entry inhibitors as "virustats" to prevent sexual transmission of HIV infection

**DOI:** 10.1186/1742-4690-5-116

**Published:** 2008-12-18

**Authors:** Michael M Lederman, Robin Jump, Heather A Pilch-Cooper, Michael Root, Scott F Sieg

**Affiliations:** 1 Department of Medicine, Case Western Reserve University, 1100 Euclid Ave, Cleveland, OH 44118, USA; 2 Kimmel Cancer Center, Thomas Jefferson University, 233 South 10th Street, Philadelphia PA, 19107, USA

## Abstract

With the continuing march of the AIDS epidemic and little hope for an effective vaccine in the near future, work to develop a topical strategy to prevent HIV infection is increasingly important. This stated, the track record of large scale "microbicide" trials has been disappointing with nonspecific inhibitors either failing to protect women from infection or even increasing HIV acquisition. Newer strategies that target directly the elements needed for viral entry into cells have shown promise in non-human primate models of HIV transmission and as these agents have not yet been broadly introduced in regions of highest HIV prevalence, they are particularly attractive for prophylaxis. We review here the agents that can block HIV cellular entry and that show promise as topical strategies or "virustats" to prevent mucosal transmission of HIV infection

## Introduction: the compelling need for prevention of HIV infection

As the pandemic spread of HIV infection and AIDS continues, there is increasing need to develop strategies for its containment. Since sexual transmission of HIV infection is the most important route of transmission throughout the world [1], approaches to limit transmission by this route are especially needed. To date, there is reason to believe that three prevention strategies work in this arena, but there are limits to their implementation. First it is a tautology that avoidance of sex will result in a decrease in sexual transmission of HIV. Despite innumerable campaigns encouraging abstinence or monogamy and some indications that some of these campaigns might have had limited effect [2], we haven't yet figured out a way to convince ourselves that avoidance of sex is better than having it when the opportunity arises. Likewise, while there is strong evidence that regular use of condoms will decrease the risk of HIV transmission by at least 80% [3], there is often resistance to their use for reasons that may relate to perceptions of pleasure, perceptions of trust and fidelity, social norms, and of access and opportunity [4]. Finally while there is strong evidence that male circumcision will decrease the risk of HIV acquisition by half or more [5-7], broad "roll-out" of circumcision has not yet been implemented. Though this is likely to be remedied soon and should have measureable impact on HIV spread, protection is not complete and additional methods of prevention will surely be needed

While a vaccine that is capable of providing sterilizing immunity to HIV is rightly the goal of intensive research, vaccine candidates plausibly capable of inducing such protection are not nearly within reach and in fact there is only limited insight into what it will take to design such candidates [8,9]. Thus there is compelling need to develop additional effective strategies for the prevention of sexual transmission of HIV.

### We should no longer develop "Microbicides" for the prevention of HIV infection

The term "microbicide" has been used to describe agents that can be applied topically to mucosal surfaces in order to prevent HIV transmission. We think that the term is both inaccurate and misleading and should not be used in polite company (at least not when discussing HIV prevention). We outline below why we would like to see this word take its rightful place beside "impact" (the verb) and the thoughtless "gerundification" of perfectly proper nouns such as "text" and "parent".

First, the most promising topical strategies to prevent HIV transmission are not microbicidal in so far as they do not kill microbes (or viruses for that matter). They achieve their effect by blocking HIV replication through interference with either a viral or a host element that is necessary for viral propagation. Second (and this is where even words can be dangerous), those agents that were in fact microbicidal (i.e., they destroyed viruses and other microbes in the test tube) have been disastrous failures in the clinic, in large part because they were broadly "microbicidal". There was early hope that topical application of a single agent might kill or otherwise render non-infectious HIV as well as a variety of other sexually transmissible pathogens. Unfortunately, the agents that had this broad killing activity were primarily soaps or detergents that dissolved the microbial cell wall or membrane. This activity was predictably toxic to human cells as the lipid membrane that surrounds the HIV capsid is always derived from the human cell in which the virions were produced. This hazard turned out to be significant in the clinic as topical application of the detergent N-9 not only failed to protect against HIV acquisition, but also likely increased infection risk as a result of toxicity to the vaginal mucosal surface [10]. A further trial of another microbicide detergent -SAVVY- nearly doubled the risk of HIV acquisition among recipients (hazard ratio 1.7), but with few events, these differences were not significant (CI = 0.9–3.5) [11]. Despite these predictably discouraging results, other detergents are still being studied with the aim of preventing HIV transmission. Such studies make us very anxious.

We would propose, therefore, that the term "microbicide" not be used when discussing HIV prevention. Instead, perhaps, a complex but more accurate phrase could be "topical prevention strategies," or even the simpler term **"virustats," **since the most promising agents effectively block HIV from replicating but do not "kill" it. This proposal may be a losing battle as there is something to be gained from branding a term and acknowledging its wide recognition by both the scientific and the lay communities. Nonetheless, one of the major advantages to writing a review article (and there are many downsides) is that you can pick your own battles and hope that others might see it your way.

### What topical prevention strategies might be implemented to prevent sexual transmission of HIV?

The viral replication cycle offers a number of opportunities for intervention to prevent HIV acquisition [12-15]. We will focus this piece on strategies that block HIV entry into cells, strategies that to us are among the most attractive for prevention. Strategies targeting later points in the viral replication cycle also are quite plausible but with some limitations as will be discussed below.

### HIV entry into host cells

**Figure 1:**
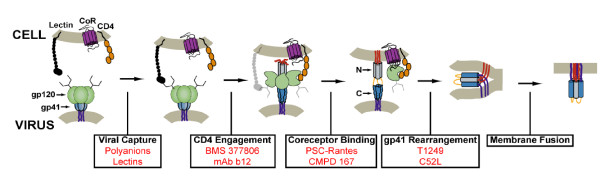
**HIV entry and its inhibition**. In the native conformation of Env, a canopy of three gp120 molecules (green) covers gp41, holding the trimer in a metastable conformation. Binding to cellular lectins (black) keeps Env close to the cellular membrane, facilitating subsequent interactions with CD4 (orange) and a chemokine receptor (CoR, purple). These gp120/receptor interactions trigger gp41 to extend and insert its N-terminal fusion peptide segment (red) into the target cell membrane. Ultimately, the exposed heptad repeat segments in the N- and C-terminal regions of the gp41 ectodomain [labeled N (gray) and C (blue)] associate to form the trimer-of-hairpins structure, juxtaposing viral and cellular membranes in a manner crucial for membrane fusion. HIV entry inhibitors investigated as potential topical prevention strategies are listed in red below each modeled transition.

HIV cellular invasion is a complicated process coordinated by the sequential binding of the viral envelope to cellular receptors (Figure 1). The responsible viral component, Env, is a heavily glycosylated, trimeric protein composed of two subunits, gp120 and gp41. Initial cellular capture of the virus is achieved through Env glycans that bind cellular lectins (DC-Sign and related C-type lectins)[16]. For some special antigen-presenting cells (mucosal dendritic cells), captured virions can be conveyed to a protected cellular compartment, enabling infectious HIV to be transported *in situ *to adjacent lymphoid tissue containing an abundance of infectable target cells [17]. On susceptible target cells, the Env-lectin interactions keep the virus close to the membrane, facilitating the binding of gp120 to its primary receptor, CD4[18]. This event induces a structural change in gp120 that exposes new surfaces capable of binding 7-transmembrane G-protein-coupled chemokine receptors (referred to as HIV coreceptors). In humans, the key coreceptors utilized by HIV are CCR5 and CXCR4, even though numerous other chemokine receptors appear to support infection *in vitro*. Interestingly, almost all cases of acute infection involve CCR5-using HIV. Consequently, individuals lacking cell surface-expressed CCR5 (owing to a 32-base pair deletion in the open reading frame of both alleles) are almost completely protected from acquiring HIV infection [19,20]. In the rare instances in which these individuals have been found to be HIV positive, the viruses were shown to utilize CXCR4 for cellular entry [21-32].

The interactions between gp120 and cellular receptors trigger a series of conformational transitions in gp41 that ultimately lead to the fusion of viral and cellular membranes. Initially, gp41 extends to insert its N-terminal "fusion peptide" segment into the target cell membrane [33]. Because gp41 already contains a transmembrane region embedded in the viral envelope, this high energy, extended conformation essentially bridges the space between target cell and virus. Driven by the tight association of heptad-repeat (HR) segments in its N- and C-terminal regions, the gp41 ectodomain eventually collapses into a compact structure known as a trimer-of-hairpins[34]. Formation of the gp41 trimer-of-hairpins brings the fusion peptide, the transmembrane region and their associated membranes into the close proximity required for the fusion process. Although evidence supports the gp120-CD4 interaction initiating gp41 structural changes, the precise role of gp120-coreceptor interaction in promoting formation of the gp41 trimer-of-hairpins remains a mystery [35,36].

### Strategies to block HIV entry into cells

Blocking the entry of HIV into target cells should be an effective mechanism to neutralize HIV infectivity since cellular infection and use of cellular machinery are necessary for production of new viruses. Entry can be blocked by targeting either the viral envelope glycoprotein, or host elements such as CD4 or CCR5.

### Inhibition of HIV replication by targeting the HIV envelope

#### Polyanions

A variety of negatively charged polyanions such as cellulose sulfate, dextran sulfate, carageenan and PRO-2000 are capable of binding to the HIV envelope and preventing cellular infection. In part because these agents are inexpensive to make and showed some *in vitro *activity, a number of them were accelerated into development as topical prevention strategies. Unfortunately, these agents tend to be far more active against the more positively charged CXCR4-using (X4-tropic) viruses than they are against the CCR5-using (R5-tropic) viruses that are responsible for most instance of HIV transmission [37,38]. In fact both dextran sulfate and cellulose sulfate can actually increase HIV infectivity *in vitro *[39,40], and plasma levels of HIV were increased in persons receiving treatment with dextran sulfate *in vivo *[41]. Thus, it is not surprising that several large placebo-controlled trials of these agents have failed to demonstrate protection against HIV infection [40]. In some instances, HIV transmission may even have been increased in persons receiving cellulose sulfate. Although one arm of a polyanion (PRO-2000) trial is still ongoing and we do not know yet whether this approach will prove useful, the poor track record of trials with other anions and the recent termination of the higher dose arm of this same PRO-2000 trial for futility tempers enthusiasm for this approach. On the other hand, the relationship between *in vitro *enhancement of infection and *in vivo *protection remains unclear. A recent study confirmed *in vitro *enhancement of infection by the polyanion carageenan but showed significant protection from SHIV vaginal challenge in rhesus macaques [42]. At the same time, this agent failed to protect women from HIV infection in a large randomized controlled clinical trial [43]. How much of this relates to the activity of the agent, the durability of its protection or the consistency of its application in the clinical trial remains to be determined. SPL 7013 is a polylysine based polyanionic dendrimer with naphthalene disulfonic acid surface groups that when formulated as a 5% gel protected 6 of 6 pigtailed macaques from infection with the predominantly CXCR4 tropic SHIV 89.6 [44]. Similar *in vitro *antiviral activity was reported against both SHIV 89.6 and the R5 tropic SHIV 163P3 [44] but to date, no *in vivo *challenge studies using an R5 tropic virus have been reported.

#### Lectins

Cyanovirin and griffithisin are small lectins that demonstrate potent *in vitro *activity against HIV replication [45,46]. They appear to bind Env glycans and interfere with cellular entry, most likely by blocking viral capture through cellular C-type lectins and/or interfering with gp120 binding to cellular CD4 and coreceptor. Cyanovirin is a 101 amino-acid protein produced by the cyanobacterium *Nostoc ellipsosporum*. Its gene has been inserted into lactobacilli that are common residents among the vaginal microbiota [47]. Sustained expression of cyanovirin by these bacteria within the vagina may provide some protection against vaginal acquisition of HIV infection, but this observation remains to be shown in a relevant animal model. Griffithisin is a 121 amino-acid protein derived from the algae Griffithsia and is currently being explored as a candidate for topical prevention of HIV transmission [48]. Immunogenicity is a potential issue for these foreign proteins after repeated topical application. Moreover, there is the additional risk that these lectins will bind to and crosslink glycoproteins present on immune cell surfaces, potentially activating these cells nonspecifically and stimulating mitogenic activity [49]. How much of a problem these concerns will be remains to be determined.

#### Monoclonal antibodies

In many viral infections, antibodies that can neutralize infectivity have the ability to provide sterilizing immunity against infection. Although neutralizing antibodies are readily demonstrable in the plasmas of persons who acquire HIV infection, these antibodies are typically type-specific and virus escapes rapidly from neutralizing activity [50,51]. A small number of human or "humanized" broadly neutralizing monoclonal antibodies (mAb) have been developed that bind to limited regions of the HIV envelope [52]. Of these, mAb b12, which recognizes the CD4 binding domain of HIV gp120, provided partial protection (9 of 12 animals protected) in the rhesus vaginal challenge model [53]. Two others, mAbs 2F5 (which targets the C-terminal region of the gp41 ectodomain) and 2G12 (which recognizes sugar moieties on gp120) also provided partial protection when co-administered systemically in combination with polyclonal anti-HIV immunoglobulin [54]. The virus utilized in these studies, however, was SHIV 89.6, which predominantly uses CXCR4 for entry. (It should be noted that in these latter studies, the antibodies were not applied topically). Such antibodies are costly to produce in large scale because they must be generated in mammalian cells capable of post-translational modification. Nonetheless, their activity *in vivo *demonstrates that targeting these regions of the viral envelope might provide a plausible topical protection strategy for a product that is simpler to make. In addition, as newer technologies are being developed to express biologically active antibodies or their fragments (see for example [55,56]), these approaches may become increasingly affordable and practicable.

#### BMS 377806

This small molecule binds to gp120 and interferes with cellular receptor interactions, thereby blocking infectivity [57]. While there is still some uncertainty as to the precise mechanism of activity of this agent, it provides substantial protection (6 of 8 animals protected) when used alone and, when used in combination with other entry inhibitors, can provide complete protection [58].

#### Inhibitors of gp41

Agents that disrupt gp41 conformational changes required for HIV membrane fusion effectively inhibit viral entry[34]. The best characterized are linear peptides originally derived from the C-terminal heptad repeat segment (C-HR) and adjacent membrane-proximal region of the gp41 ectodomain [59,60]. These so-called C-peptides bind the gp41 N-terminal heptad repeat segment (N-HR) exposed in the extended, prehairpin intermediate conformation of the ectodomain [34]. Once bound, they block association of N-HR and C-HR segments, thereby disrupting trimer-of-hairpins formation and inhibiting viral membrane fusion. While C-peptides are only effective during a short kinetic window between CD4-gp120 binding and trimer-of-hairpins formation, they can possess potent (low nanomolar) antiviral activity *in vitro*. With parenteral administration of 90 to 180 mg/day, the 36-mer C-peptide enfuvirtide (T-20) can effectively suppress HIV replication in humans and is currently used as a salvage therapy for HIV-1 infected individuals [61] Surprisingly, the effectiveness of enfuvirtide as a topical protective agent against mucosal HIV transmission has not been reported. However, the second generation agent T-1249 and an extended C-peptide C52L have been shown to effectively reduce HIV mucosal transmission in macaque studies [62,63]. As a monotherapy, T-1249 completely protected against infection following vaginal SHIV challenge, but only at concentrations in excess of 100 μM. For C52L, concentrations as high as 1.5 mM only afforded protection in 60% of challenged animals. However, the activity of C52L is enhanced in combination with other antiviral agents (especially inhibitors of coreceptor binding), consistent with the synergistic activity between C-peptides and different HIV entry inhibitors observed *in vitro *[64,65]. As with cyanovirin, mucosal bacteria secreting C-peptides have been generated, but whether colonization with these microorganisms can protect against HIV transmission remains to be tested [66].

The 5 order-of-magnitude disparity in C-peptide potencies determined from *in vitro *infectivity experiments and from vaginal challenge studies is disconcerting, and its cause unknown. Similar differences are also observed for entry inhibitors of different classes, leading many investigators to speculate that these observations reflect some intrinsic difficulty in delivering inhibitors to sites where they need to act [67]. Two aspects of C-peptide inhibition compound this problem. First, C-peptides target an intermediate state during the entry process and, thus, must be present at high levels at the site of viral infection[34]. They do not bind the Env native state prior to CD4 interaction, and they do not work on target cells. Where in the vaginal mucosa HIV first encounters and infects target cells remains unknown, but such events are likely to take place in the deep epithelium or submucosa. Hence, to effectively block all mucosal transmission, C-peptides must passively diffuse a significant distance through the vaginal epithelium and surrounding tissue. Second, C-peptides are unstructured in solution and readily susceptible to proteolysis [68]. The vaginal milieu and surrounding tissues are full of proteases that can potentially limit the bioavailability these antiviral agents. To date, the search for non-peptide, small molecule gp41 inhibitors has not yielded compounds with sufficient antiviral potency. Recently, however, protease-insensitive peptides composed of d-amino acids (D-peptides) have been developed that target a small region of the N-terminal HR segment [69,70]. Crosslinked versions of these D-peptides show potent, broad-spectrum inhibitory activity and represent promising candidates for a future topical antiviral strategy.

### Inhibition of HIV replication by targeting host cell surface receptors

#### Blockade of CD4

In principal, blockade of CD4 by targeting the envelope binding domain of the HIV receptor should decrease HIV infectivity and one humanized monoclonal antibody, TNX-355 (Ibalizumab) has demonstrated antiretroviral activity in HIV infected persons after systemic administration [71], however there do not appear to be plans to develop this reagent as a topical prevention strategy.

#### Blockade of CCR5

Since the original discovery that CCR5 and CXCR4 are critical to HIV entry [72-78], major effort has been devoted to developing reagents that target these coreceptors and disrupt their interactions with gp120 [13,79]. To date, efforts to develop CCR5 inhibitors have been much more successful than strategies targeting CXCR4. From the standpoint of a topical prevention strategy, CCR5 inhibition appears to be much more important as almost all cases of new infection are caused by R5-tropic viruses. This point is underscored by the rarity of HIV infected individuals who lack surface-expressed CCR5. Nonetheless, there was no certainty that mucosal blockade of CCR5 would be sufficient to provide protection against HIV transmission. Viruses captured by DC-SIGN (or related C-type lectins) on certain submucosal dendritic cells might be transported and presented for trans-infection to CD4^+ ^CCR5^+ ^immune cells found deeper within the body. Thus it was important to learn that topical blockade of CCR5 was sufficient to provide very high level [58] or complete [80] protection against vaginal transmission of the R5 tropic SHIV 16P3.

There are now three strategies to block HIV replication by targeting CCR5. Humanized monoclonal antibodies have been developed that bind to CCR5 and block its interaction with the HIV envelope. Both HGS 004 and PRO 140 have been given systemically to persons with HIV infection and have demonstrable antiretroviral activity *in vivo *[81,82]. Neither is being developed for topical application. A number of small molecule allosteric inhibitors of CCR5 have been developed for systemic administration; one of these, maraviroc, has been approved for treatment of HIV infection, and another, vicriviroc, has demonstrated *in vivo *efficacy [83,84]. A third small molecule CCR5 inhibitor, CMPD 167 (Merck), is not being developed for systemic administration but has shown protective activity in the rhesus vaginal challenge model [58]. The advantages to developing these small molecule CCR5 inhibitors as topical agents to prevent HIV infection include 1) the rigorous safety testing that they have undergone during trials of systemic administration, 2) the fact that they are relatively inexpensive to produce and 3) their lack of agonist activity on the chemokine receptor. In fact, these molecules block the agonist activity of natural chemokine ligands and, thus, are expected to be anti-inflammatory. Conceivably, this property might protect the vaginal mucosa from the inflammation caused by concurrent bacterial vaginosis [85] or sexually transmissible infections [86] that have been linked to enhanced risk for HIV acquisition.

A third mechanism by which CCR5 can be targeted to block mucosal HIV transmission is by application of their natural or modified chemokine ligands. By experimental modification of the amino terminus of RANTES, Robin Offord and Oliver Hartley have developed a series of RANTES analogues with substantially greater antiretroviral activity than the native molecule [87]. The first lead molecule to be tested in the non-human primate vaginal challenge model, PSC-RANTES, has several non-natural amino acid residues of the amino terminus which are responsible for its antiretroviral activity at sub-nanomolar concentrations. PSC-RANTES is an agonist of CCR5 and induces durable (at least 24 hrs) internalization of CCR5, rendering the coreceptor inaccessible to the viral envelope [87]. When tested in the rhesus vaginal challenge model, PSC-RANTES completely protected 12 of 12 animals from infection with SHIV 162P3 ([80] and Veazey, unpublished). Because the amino terminus of PSC- RANTES comprised non-natural amino acids, the molecule cannot be produced completely by biosynthesis, rendering cost of chemical synthesis an insurmountable obstacle for large scale production. For this reason, a novel phage display selection method was applied to develop recombinant RANTES variants with high level potency [88]. The selection strategy successfully identified two fully recombinant molecules, 5P12 and 6P4 RANTES, with the comparable *in vitro *antiviral potency to that of PSC-RANTES [89]. Morevoer, both fully recombinant molecules completely protected 5 of 5 challenged monkeys from SHIV 162P3 infection in the rhesus vaginal challenge model [90]. These new molecules are stable at high temperatures and are resistant to loss of biologic activity after exposure to vaginal and seminal fluids [91]. Because they are fully recombinant, it should be possible to produce them in sufficient bulk and affordably for distribution in the developing world where these agents are needed most. Interestingly, while having equipotent antiviral activity *in vitro *and *in vivo*, these two new molecules have distinctly different mechanisms of action. Similar to PSC-RANTES, 6P4-RANTES is a CCR5 agonist that activates cell signaling cascades and receptor internalization after binding. By contrast, 5P12-RANTES binding fails to properly activate the receptor (no observed changes in intracellular calcium levels) and does not promote CCR5 internalization. It remains to be seen whether simple receptor occupancy without cellular activation will prove a better topical prevention strategy than durable coreceptor internalization with induced cellular activation. Perhaps the ideal strategy will be one that promotes durable receptor internalization without signal transduction, and development of such a molecule may be achievable. Other G-protein-coupled receptors can be internalized by internalized by certain ligands without demonstrable agonist activation [92-95]. Already, one chemokine analog, 5P14-RANTES [96], has been identified that is as potent as PSC-RANTES, does not stimulate Ca2+ flux, but induces the sequestration of CCR5 with approximately half the efficiency of PSC-RANTES and 6P4-RANTES.

#### Combination strategies

Combination antiretroviral therapies have proven enormously successful in the treatment of established HIV infection. In fact, single agent strategies have invariably failed, in large part due to the predictable emergence of viruses that contain mutations conferring resistance to the antiviral drug. Though the principles are not exactly the same, there are solid rationales to the development of combination strategies for the topical prevention of HIV mucosal transmission. First, while emergence of resistance is unlikely to occur upon HIV exposure during sex with an infected partner, the increasing transmission of drug resistant viruses among newly infected persons [97,98] may render some topical prevention strategies less effective (see below). Thus combinations of agents that disrupt different elements of the HIV life cycle may also provide a broader safety net of protection. Second, additive or synergistic activities might enhance the level of protection against the establishment of infection. Using SHIV 162P3 in a macaque vaginal challenge model, Veazey *et al*. have shown that combinations of agents that block viral entry provide better levels of protection than any single agent used alone [58]. While CMPD 167, C52L, BMS 378806 failed to completely protect challenged animals alone, combinations of two and all three of these agents proved fully protective. Unfortunately, before a single agent is proven to be safe and effective, there are practical (e.g. formulation) and registrational hurdles to the development of combinations of active agents as topical prevention strategies. We do not believe these hurdles to be insurmountable. Formulations compatible with multiple active agents can be developed and agencies charged with oversight of drug development can work closely with investigators to design and accelerate the pace of studies designed to test the safety and activity of plausible combination prevention strategies.

### Additional comments

As has been stated by nearly every researcher in the field, a successful "microbicide" or topical prevention strategy must be safe, effective and affordable. There are currently a good number of promising candidates under development, including inhibitors of entry as well as inhibitors of other elements of the viral life cycle[12]. Unfortunately, the field (and public perceptions of the field) has been considerably damaged by a number of widely publicized dramatic failures of large test of efficacy studies, some of which have actually increased the risk of HIV transmission (reviewed in [99]). While the urgent need to halt the spread of the epidemic is real, the field needs to be more selective about which agents to bring forward into large efficacy studies. Candidates for topical prevention strategies must have a plausible chance for succeeding. Thus, they must be effective in preclinical studies that include protection of non-human primates from infection with an R5 tropic virus. The agents must be safe. Thus, they must be well tolerated after multiple applications in women who are at low risk for HIV infection and must not induce the inflammation that has been linked to an increased risk for HIV transmission. Ideally, they should not perturb the normal vaginal microbiota, nor increase risks for acquisition of other sexually transmissible infections. Finally, these drugs should have durable activity. Analyses from a failed prevention trial suggest that agents requiring application just prior to coitus are not likely to be used often enough to provide sufficient protection [100]. Thus, only strategies that provide substantial protection from infection for at least a full day or even longer are likely to prove effective in clinical settings. It remains to be seen whether durable protection can be provided by formulation in slow release gels or films or by application using vaginal rings that have been used for slow release of contraceptives[101,102].

It may be problematic to apply a strategy for topical prevention in a community that is using the same strategy for treatment. First, the predictable emergence of resistance during therapy may render that strategy less effective when applied to prevent the initial acquisition of infection. Second, topical application of an antiviral drug by persons who are unknowingly infected may provide sufficient selection pressure systemically or even at mucosal and peri-mucosal sites of HIV replication to induce emergence of resistance, rendering subsequent treatment less effective. How much of an issue these concerns will be remains to be seen, but they provide some rationale for the need to develop different strategies for treatment and for prevention. Lastly, a major rate limiting step to the implementation of a topical HIV prevention strategy is the cost of development. The cost can be measured both in real dollars and in expertise. Most scientists engaged in this new area of prevention research are not experts in drug development. Those with the managerial and scientific expertise to bring a new drug to the clinic are found in the pharmaceutical industry, which, for the most part, has steered clear of topical HIV prevention strategies for market reasons. Getting these companies engaged in a substantive way might help accelerate the process.

## Competing interests

The authors declare that they have no competing interests.

## Authors' contributions

MML, RJ, MR and SFS contributed to the original drafts of the manuscript. All authors critiqued drafts of the manuscript and all authors read and approved the final version.
